# The Association between Living Status Transitions, Behavior Changes and Family Relationship Improvement among Methadone Maintenance Treatment Participants in Guangdong, China

**DOI:** 10.3390/ijerph17010119

**Published:** 2019-12-23

**Authors:** Shaokun Liu, Xia Zou, Xiaoling Huang, Yin Liu, Qian Lu, Li Ling

**Affiliations:** Department of Medical Statistics, School of Public Health, Sun Yat-sen University, Guangzhou 510080, China; liushk5@mail2.sysu.edu.cn (S.L.); zouxia@mail3.sysu.edu.cn (X.Z.); huangxling25@mail.sysu.edu.cn (X.H.); liuy429@mail2.sysu.edu.cn (Y.L.); luqian7@mail2.sysu.edu.cn (Q.L.)

**Keywords:** methadone maintenance treatment, family relationship, living status transitions, behavior changes

## Abstract

The quality of family relationships is important for individual and family well-being. Improving family relationships is also an important goal in methadone maintenance treatment (MMT). Little is known about factors associated with the improvement of family relationships among MMT clients. This study aimed to identify factors associated with family relationship improvement in MMT. We retrospectively analyzed existing data from 2006 to 2014 at 15 MMT clinics in Guangdong, China, including 2171 subjects with 4691 follow-ups. Generalized estimating equations were used to investigate the association between living status transitions, behavior changes and family relationship improvement, with covariates controlled for. Family relationship improvement was found in 23.1% of all follow-up intervals. Participants who began living with family, living on a regular wage, and gained employment were more likely to have improved family relationships. The quality of family relationships also improved among participants who ceased contact with drug-addicted fellows, ceased drug use, and those who were sexually active. These results suggest that improvement in living status, positive changes in drug use, and sexual activity are associated with family relationship improvement and corresponding interventions may be developed to facilitate clients’ recovery.

## 1. Introduction

The association between social relationships and health has been long investigated [1,2,3]. Family is the basic unit of society, and family relationships constitute an important part of social relationships. The quality of family relationships has significant impact on an individual’s and family’s well-being across the life course [4,5,6]. Empirical studies have provided compelling evidence linking a lower quality of family relationships with worse mental health, worse outcomes of chronic disease, and lower utilization of health services [7,8,9,10,11]. The family also serves as the earliest context for individual development, so a strong family relationship is an important guarantee of family functions to support individual physical and mental growth [12,13,14]. Therefore, family relationships are often involved in health promotion.

In the treatment of opioid dependence, family is an integral part of recovery. People who are addicted to drugs often have poor relationships with their family and are isolated by family members, especially in many Asia countries where the notion of family is extremely valued. They can neither get support from family nor play their role within the family, which can lead to an erosion of the ideal family function. The recovery of healthy family relationships will not only facilitate the normal functioning of the family, but also help an individual abstain from drugs.

Methadone maintenance treatment (MMT) is an effective treatment for opioid dependence [15,16]. The quality of family relationships is also an important outcome of MMT effectiveness. For example, in Myanmar, according to their MMT guidelines, integrating clients back into their family is one of the goals of MMT and it is also an indicator for complete cessation of MMT [17]. In the Chinese MMT program, quality of family relationships is one of the outcomes regularly assessed during treatment [18]. Previous studies have shown that most clients improved their family relationship after commencing MMT [19,20,21]. A meta-analysis of 28 studies on family relationships in China showed an increase in the proportion of participants having a good family relationship after 6 months (59.6%), 12 months (75.0%), and >12 months (83.2%), compared with baseline (37.9%) [22].

A wide range of factors have been identified in previous studies which are associated with the quality of family relationships. Some sociodemographic factors such as educational level were found to be associated with relationship quality. Results from Surveys of Income and Program Participation (SIPP) (U.S. Census Bureau, 2001) showed that marital dissolution rates fell among women with a 4 year college degree or more, but remained high among women with less than a 4 year college degree [23]. The result was furtherly supported by Esping-Andersen’s thesis [24]. Family economic factors can also play an important role in relationship quality [25]. Conger et al. surveyed 76 white, middle-class couples from a rural midwestern county in America and found that economic strain increased the hostility and decreased the warmth/supportiveness of husbands toward their wives, and had an indirect effect on marital quality through the husband’s behaviors [26]. In addition, personal traits and behaviors have also been observed as correlates of family relationship. Results of a national survey conducted among 250 families in Ireland indicated that the ability to resolve arguments and women’s positive emotionality positively associated with the quality of couple relationship [27]. Another study conducted among spouse caregivers of patients with dementia showed that deterioration of relationship quality perceived by caregiver was specifically associated with the presence of behavioral problems, notably apathy of patients [28]. Other factors associated with family relationship quality included factors of family structure (e.g., family type, family size, and residential status), lifestyle, and genetic and environmental factors [6,29,30].

However, most of these studies were cross-sectional studies. They did not examine the change of family relationship quality. It is not clear which factors associated with the change in family relationship quality. In some relationship education programs or therapy for improving relationships, researchers have proposed basic models to explain the mechanisms of relationship change with some validity based on social learning and cognitive behavioral theory. Doss et al. and Rauer et al. both reported a significant correlation between changes in behaviors and changes in couples’ relationships before and after an intervention that aimed to improve couple relationship [31,32]. That meant an increase in positive behaviors and a decrease in negative behaviors were related to the improvement of the relationships. In another couple relationship education program, changes in parent–child relationship quality were associated with changes in parents’ conflict-related behaviors and mediated the associations between changes in couples’ relationship quality and changes in a child’s mental health symptoms [33]. These studies focused on the improvement of relationship quality, while they were all conducted among general population.

To ensure the long-term success of MMT programs and inform efforts to improve family relationship quality of MMT clients, there is a need to find out the underlying mechanisms for improving family relationships during MMT. Unlike previous studies which assessed status and behavior at a single moment in time, this study focused on the process of changes during MMT and explored the association between living status transitions, behavior changes and family relationship improvement.

## 2. Materials and Methods

### 2.1. Study Design and Setting

This retrospective analysis used data from 2006 to 2014 from 15 MMT clinics in Guangdong province, China. Guangdong Province is located in the southern coast of China and has the largest number of drug abusers in China [34]. The government established 63 MMT clinics in Guangdong by January 2016. In this study, we approached 15 MMT clinics located in nine cities of Guangdong province and obtained consent from all the clinics. All of the clinics in this study were established between 2006 and 2008.

### 2.2. Data Collection

Demographic information, drug use history, and HIV/HCV infectious status were recorded during MMT enrollment. Family relationship, living status, and behavioral information was collected using a uniform structured questionnaire at baseline and follow-ups at 6 months, 12 months, and annually thereafter. MMT clients were also required to submit a urine sample at random each month for opioid testing. Daily medication information (including dosage of methadone and date of methadone intake) was recorded. All the data were documented in the Chinese National MMT Data Management System. We collected the records from 1 January 2006 to 12 March 2014. All client data were de-identified before analysis.

### 2.3. Study Participants

The participant eligibility criteria were (1) registered in one participating MMT clinic in this study; (2) had baseline information; (3) had at least one follow-up record before their first dropout (failing to take methadone for 14 consecutive days or more was considered as a dropout [35,36,37]).

### 2.4. Measures

#### 2.4.1. Family Relationships

Participants were asked “How is your current relationship with your family” to measure the quality of overall family relationships at baseline and each follow-up. Three quality levels were defined: good (close affective relationships with family members), general (moderate affective relationships with family members), and difficult (no affective relationships with family members) [38].

#### 2.4.2. Living Status

Living status measurements included (1) current residential status, measured by asking “with whom do you live” (1 = living with families or relatives, 2 = living alone, 3 = living with friends, 4 = others), (2) main source of living costs over the past six months (1 = living off family members or friends, 2 = regular wages, 3 = temporary wages, 4 = other), and (3) current employment status (1 = temporary work or fixed work, 2 = unemployed).

#### 2.4.3. Drug Use and Sexual Behaviors

Behaviors in this study included (1) contact with drug-addicted fellows over the past month, measured by asking “how many times do you have contact with other drug users”, (2) concurrent drug use over the past month, defined as having a positive result in the most recent urine test or self-reported concurrent drug use behavior in the past month (1 = yes, 2 = no), (3) having sexual activity over the past three months (1 = yes, 2 = no).

#### 2.4.4. MMT-Related Characteristics

Similar to previous research, we defined the following treatment related variables:

1. Dosage of methadone, defined as the median daily dosage of an MMT participant during a follow-up interval;
2. Attendance rate, defined as the number of days a participant actually received a methadone dose to the total days of a follow-up interval;
3. Urine test participation rate, calculated by dividing the times of tests a participant actually attended by the total times of tests one should attend during a follow-up interval;
4. Urine positive rate, calculated by dividing the times of positive results by the total times of tests one actually attended during a follow-up interval.

### 2.5. Dependent Variable

Our primary outcome of interest was family relationship improvement among MMT participants. At each follow-up, participants were considered to have ”improved family relationships” if they reported a better level of family relationships (i.e., from difficult to general or good, from general to good) during this follow-up interval.

### 2.6. Independent Variables

This study focused on the living status transitions and behavior changes, which were identified during each follow-up interval if the presence or absence of a relevant variable (living status or drug use and sexual behaviors) differed from the previous one. The transitions included (1) transition of residential status: began living with family (i.e., from living with drug-addicted fellows, others, or living alone to living with family); ceased living with family; unchanged; (2) transition of the main source of living costs: began living on a regular wage (i.e., from living off family or friends, a temporary wage, or other, to having a regular wage); lost a regular wage; unchanged; (3) transition of employment status: gained employment (i.e., from being unemployed, temporary work to stable work); lost employment; unchanged; (4) change of contact with drug-addicted fellows: began contact, ceased contact, unchanged; (5) change of concurrent drug use behaviors: began drug use, ceased drug use, unchanged; (6) sexual behavior changes: were sexually active (from “inactive” to “active”); were sexually inactive (from “active” to ”inactive”); unchanged. For example, if an individual was unemployed at baseline and employed at 6 months but then had no employment at 1 year and subsequent years, the independent variable, that is, transition of employment status, would be “gained employment” at 6 months and “lost employment” at 1 year and “unchanged” at subsequent years. Other independent variables were addressed in the same way.

We controlled following covariates for potential confounders: (1) demographic characteristics including gender, age, ethnicity, educational level, and marital status; (2) drug use history including the age of initial drug use, years of drug use before enrollment in MMT, and injection drug use history in the six months prior to MMT; (3) Infection status of human immunodeficiency virus (HIV) and hepatitis C virus (HCV) at baseline; (4) MMT and urine test characteristics during follow-up intervals, including dosage of methadone; attendance rate; urine test participation rate; urine positive rate; length of follow-up intervals (months).

### 2.7. Statistical Analysis

All analyses were conducted using SAS 9.4 software (SAS Institute Inc., Cary, NC, USA). Categorical variables were described by frequency and proportion, and numerical variables were described by mean and standard deviation (SD) or median and interquartile range (IQR). Considering the correlation of repeated measurements with one individual, we used generalized estimating equations (GEE) for binary outcomes to investigate the association between living status transitions, behavior changes and family relationship improvement, with covariates controlled for. Logit link function and exchangeable correlation structure were used to model the probabilities of family relationship improvement. Independent variables with a *p* value of less than 0.2 in a univariable GEE analysis were included in the multivariable GEE analysis. The odds ratios (OR) with 95% confidence intervals were reported in the GEE analysis. All reported *p*-values were two-sided and considered significant with a *p* < 0.05.

### 2.8. Ethical Statemtent

This research was approved by Sun Yat-sen University’s School of Public Health’s Institutional Review Board (No: 2013-26).

## 3. Results

### 3.1. Characteristics of Participants in This Study

Between Jan 01, 2006 and March 12, 2014, 2171 eligible participants with a total of 4691 follow-ups were included in this study. Participants in this study on average were (36.5 ± 6.6) years old, predominantly male (88.8%), Han ethnicity (98.9%), and educated to middle school and above (81.2%). About half (48.1%) were married. Participants began drug use at a median age of 23 (IQR: 20–27) years old. Most (77.8%) injected drugs six months prior to MMT. At enrollment, 5.2% of the participants were infected with HIV and 78.9% with HCV (Table 1).

### 3.2. Living Status Transitions, Behavior Changes

Family relationship improvement was found in 23.1% of the follow-up intervals. Of all the follow-up intervals, 6.7% began living with their family, 14.6% began living on a regular wage, and 20.9% gained employment during the intervals. While, 27.8% of the participants ceased contact with drug-addicted fellows, 36% ceased drug use, and 14.7% reported being sexually active during a follow-up interval (Table 2).

### 3.3. Association between Living Status Transitions, Behavior Changes and Family Relationship Improvement

Compared with unchanged statuses, family relationship improvement was positively associated with those who began living with family (OR = 1.51, 95% CI: 1.16–1.96, *p* = 0.002), living on a regular wage (OR = 1.36, 95% CI: 1.12–1.65, *p* = 0.002), and gaining employment (OR = 1.56, 95% CI: 1.32–1.85, *p <* 0.001). While loss of employment (OR = 0.73, 95% CI: 0.56–0.93, *p* = 0.013) was negatively associated with family relationship improvement. Participants who ceased contact with other drug users (OR = 1.84, 95% CI: 1.57–2.17, *p <* 0.001), ceased drug use (OR = 1.22, 95% CI: 1.03–1.43, *p* = 0.019), and were sexually active (OR = 1.67, 95% CI: 1.38–2.02, *p <* 0.001) during the follow-up intervals were more likely to have improved family relationships. While, beginning drug use (OR = 0.65, 95% CI: 0.46–0.91, *p <* 0.013) was negatively associated with family relationship improvement (Table 3).

## 4. Discussion

There were some participants who improved their family relationships during MMT. This study, to our knowledge, is the first study collectively focused on the transitions of living status, changes in behaviors, and family relationship improvement of MMT clients. The results of this study have begun to address the factors associated with family relationship improvement in MMT by examining a model of changes based on follow-up data during treatment.

Our study indicated that improvement in living status was associated with family relationship improvement. When participants began living with family, the quality of their family relationships was more likely to improve. This is supported by previous research which suggested that family homes provide living circumstances with emotional and instrumental support to their relatives [39,40]. Living in a family home, people spend more time together, attend more family activities, and feel closer to them. Compared with those living out of the family home, people living with family reported a better sense of well-being [41]. This may facilitate a more positive view of family relationships. The quality of family relationships was also easier to improve when participants were living on a regular wage, as well as for those who gained employment. Due to the high unemployment rate among drug users, most of them live off family, friends, temporary wages, or social welfare [42,43]. Making an income illegally is also prevalent among heroin users [44]. These not only increase economic pressures on their family members but also bring shame on their family, having a further negative impact on the relationship and the user. Our results suggested that employment is closely related to rebuilding family relationships, this claim is supported by previous studies focusing on employment as an important factor in the successful recovery of individuals with drug dependence [45]. This may be explained by the abstinence from drugs and self-esteem promotion through meaningful work [46,47].

Our study also found that positive changes in drug-use and sexual behavior were associated with family relationship improvement. Participants who ceased drug use and contact with drug-addicted fellows were more likely to improve family relationships. Drug-use behaviors are stigmatized in most countries, and people who use drugs are often marginalized and isolated, even by their own family. Despite the attendance of MMT, many clients concurrently use drugs [21]. This can be a barrier to reacceptance by family members. A previous study also indicated that clients with a social network including members using drugs were more likely to concurrently use drugs [48]. Getting out of drug-addicted social relationships can not only help avoid relapse triggers but can also deliver a signal to their family that they are free from drugs in a turn to bring a more positive view of their relationships. Being sexually active during treatment was also associated with the improvement of participants’ family relationships in this study. Sexual well-being is an important part of psychological well-being and quality of life [49]. Healthy and safe sex helps to increase partner intimacy and improve relationship quality. Unfortunately, we did not differentiate different types of family relationships, and sexual partners in this study, so caution is required when interpreting such results.

In this study, living status improvement and positive changes in drug-use and sexual activity were identified to be strong correlates of family relationship improvement. However, according to our data, the quality of family relationships remain to be improved among most of MMT clients. Many of them do not have a job and cannot live on a regular wage for a long time. Continued drug use is also very common among MMT clients. Given this, combining with our initial results, we propose the following suggestions. One, the results suggested that more connections with family members provided more likelihood of promoting family relationships. Opioid dependence treatment needs the joint effort of the individual, clinic, and family. More family involvement in MMT should be taken into consideration in the future. Two, employment support should be provided to MMT clients. The MMT clinic can become a platform providing integrated services including job consultations for clients. Recently, a kind of employment intervention called therapeutic workplace showed the effectiveness in maintaining employment through an incentive program model [50,51]. However, more interventional studies are needed to verify its generalizability. Three, more strict urine tests should be administered to reduce concurrent drug use during treatment. Some incentive measures based on the urine test results may motivate clients to remain abstinent. Exploring novel strategies to help clients establish new social networks and live away from drug-addicted fellows will help them to reintegrate into the family and society. High-quality health education should be given to MMT clients to reduce the high risk of sexual behaviors. Finally, whilst our study suggests a link between living status transitions, behavior changes and family relationship improvement, further work is required to provide more evidence for their relationships. For instance, interventional study may be conducted to find out whether there is a causal link between living status improvement, positive behavior changes and family relationship improvement or MMT causes them independently.

Several limitations to this study need to be acknowledged. One, we limited the study period up to March 12, 2014 to ensure adequate sample size and the consistency of study period between all the 15 clinics. As a result, the generalizability of our findings may be slightly weakened. Two, we are unable to infer the causal relationship between living status transitions, behavior changes and family relationship improvement because they occurred during the same follow-up intervals, without knowing which occurred first. Nevertheless, these findings help verify appropriate targets for intervention.

## 5. Conclusions

This study retrospectively analyzed data from 15 MMT clinics in Guangdong, China to explore the association between living status transitions, behavior changes and family relationship improvement among MMT clients. The results showed that improvement in living status and positive changes in drug-use and sexual activity were associated with family relationship improvement. This may help us to understand how to improve MMT clients’ family relationships and to develop corresponding interventions to facilitate clients’ recovery.

## Figures and Tables

**Table 1:** Characteristics of methadone maintenance treatment participants included in this study (*n* = 2171).

Characteristics	*n* (%)
Demographic characteristics	
Gender	
Female	243 (11.2)
Male	1928 (88.8)
Age, mean ± SD	36.5 ± 6.6
Ethnicity	
Han	2148 (98.9)
Minorities	23 (1.1)
Educational level	
Primary school and below	409 (18.8)
Middle school and above	1762 (81.2)
Marital status	
Unmarried	935 (43.1)
Married	1045 (48.1)
Divorced/widowed/others	191 (8.8)
Drug use history and HIV/HCV infection	
Age of initial drug use, median (Q1, Q3)	23.0 (20.0, 27.0)
Years of drug use, median (Q1, Q3)	13.0 (9.0, 16.0)
Intravenous drug use	
No	482 (22.2)
Yes	1689 (77.8)
HIV infection at baseline	
No	2058 (94.8)
Yes	113 (5.2)
HCV infection at baseline	
No	459 (21.1)
Yes	1712 (78.9)

SD, standard deviation; Q1, lower quartile; Q3, upper quartile.

**Table 2:** Family relationship improvement, living status transitions, behavior changes, and methadone maintenance treatment (MMT) related characteristics during follow-up intervals (*n* = 4691).

Characteristics	*n* (%)
Family relationship improvement	
Improved	1085 (23.1)
Not improved	3606 (76.9)
Living status transitions	
Residential status	
Unchanged	4105 (87.5)
Living with family	3707 (79.0)
Not living with family	398 (8.5)
Began living with family	315 (6.7)
Ceased living with families	271 (5.8)
Main source of living cost	
Unchanged	3572 (76.1)
Living on a regular wage	609 (12.9)
Not living on a regular wage	2963 (63.2)
Began living on a regular wage	685 (14.6)
Lost a regular wage	434 (9.3)
Employment status	
Unchanged	3142 (67.0)
Employed	1601 (34.1)
Not employed	1541 (32.9)
Gained employment	981 (20.9)
Lost employment	568 (12.1)
Behavior changes	
Contact with drug-addicted fellows	
Unchanged	2908 (62.0)
Contacting with drug-addicted fellows	1155 (24.6)
Not contacting with drug-addicted fellows	1753 (37.4)
Began contact	481 (10.3)
Ceased contact	1302 (27.8)
Concurrent drug use	
Unchanged	2659 (56.7)
Using drugs	1262 (26.9)
Not using drugs	1397 (29.8)
Began drug use	341 (7.3)
Ceased drug use	1691 (36.0)
Sexual behavior	
Unchanged	3256 (69.4)
Having sexual activity	1977 (42.1)
Not having sexual activity	1279 (27.3)
Were sexually active	690 (14.7)
Were sexually inactive	745 (15.9)
MMT-related characteristics during follow-up intervals	
Dosage of methadone ^a^	
<30 mg	518 (11.0)
30–60 mg	2159 (46.0)
60–100 mg	1693 (36.1)
≥100 mg	321 (6.8)
Attendance rate ^b^	
<50%	263 (5.6)
50%–80%	952 (20.3)
≥80%	3476 (74.1)
Urine test participation rate ^c^	
<50%	325 (6.9)
50%–80%	982 (20.9)
≥80%	3384 (72.1)
Urine positive rate ^d^	
<20%	2434 (51.9)
20%–60%	1396 (29.8)
≥60%	861 (18.4)
Length of follow-up intervals (month), median (Q1, Q3)	6.7 (6.0, 12.2)

^a^, defined as median daily dosage of an MMT participant during a follow-up interval; ^b^, defined as the ratio of the number of days a participant actually received a methadone dose to the total days of a follow-up interval; ^c^, calculated by dividing the times of tests a participant actually attended by the total times of tests one should attend during a follow-up interval; ^d^, calculated by dividing the times of positive results by the total times of tests one actually attended during a follow-up interval.

**Table 3:** GEE analysis exploring the association between living status transitions, behavior changes and family relationship improvement (*n* = 2171).

Variables	Univariable		Multivariable	
	OR (95% CI)	*p* Value	aOR (95% CI)	*p* Value
Living status transitions				
Residential status				
Ceased living with family	0.98 (0.72, 1.32)	0.881	1.00 (0.73, 1.36)	0.999
Began living with family	1.50 (1.17, 1.93)	0.001	1.51 (1.16, 1.96)	0.002
Unchanged	1.00		1.00	
Main source of living cost				
Lost a regular wage	0.66 (0.50, 0.87)	0.003	0.83 (0.62, 1.09)	0.176
Began living on a regular wage	1.70 (1.42, 2.04)	<0.001	1.36 (1.12, 1.65)	0.002
Unchanged	1.00		1.00	
Employment status				
Lost employment	0.68 (0.53, 0.87)	0.002	0.73 (0.56, 0.93)	0.013
Gained employment	1.91 (1.62, 2.23)	<0.001	1.56 (1.32, 1.85)	<0.001
Unchanged	1.00		1.00	
Behavioral changes				
Contact with drug-addicted fellows				
Ceased contact	2.19 (1.88, 2.55)	<0.001	1.84 (1.57, 2.17)	<0.001
Began contact	0.94 (0.73, 1.21)	0.619	0.96 (0.73, 1.24)	0.736
Unchanged	1.00		1.00	
Concurrent drug use				
Ceased drug use	1.59 (1.37, 1.84)	<0.001	1.22 (1.03, 1.43)	0.019
Began drug use	0.60 (0.43, 0.84)	0.003	0.65 (0.46, 0.91)	0.013
Unchanged	1.00		1.00	
Sexual behavior				
Were sexually inactive	1.01 (0.83, 1.23)	0.930	0.94 (0.77, 1.15)	0.560
Were sexually active	1.79 (1.49, 2.15)	<0.001	1.67 (1.38, 2.02)	<0.001
Unchanged	1.00		1.00	
Demographic characteristics				
Gender				
Male	1.17 (0.94, 1.45)	0.170	1.16 (0.93, 1.45)	0.194
Female	1.00		1.00	
Age	0.99 (0.98, 1.00)	0.151	1.00 (0.99, 1.01)	0.800
Ethnicity				
Minorities	0.83 (0.45, 1.53)	0.548		
Han	1.00			
Educational level				
Middle school and above	0.97 (0.83, 1.13)	0.674		
Primary school and below	1.00			
Marital status				
Married	1.01 (0.88, 1.15)	0.934		
Divorced/widowed/others	1.04 (0.83, 1.31)	0.720		
Unmarried	1.00			
Drug use history and HIV/HCV infection				
Age of initial drug use	1.00 (0.99, 1.01)	0.437		
Years of drug use	1.01 (0.99, 1.02)	0.338		
Injecting drug use				
Yes	1.24 (1.06, 1.45)	0.006	1.10 (0.92, 1.31)	0.282
No	1.00		1.00	
HIV infection at baseline				
Yes	1.21 (0.93, 1.57)	0.162	1.21 (0.92, 1.60)	0.175
No	1.00		1.00	
HCV infection at baseline				
Yes	1.29 (1.11, 1.51)	0.001	1.19 (1.00, 1.42)	0.046
No	1.00		1.00	
MMT-related characteristics during follow-up intervals				
Average dosage of methadone				
<30 mg	1.20 (0.87, 1.66)	0.276	1.16 (0.83, 1.62)	0.389
30–60 mg	1.22 (0.92, 1.61)	0.166	1.16 (0.87, 1.55)	0.324
60–100 mg	1.28 (0.97, 1.71)	0.086	1.24 (0.93, 1.66)	0.149
≥100 mg	1.00		1.00	
Attendance rate				
≥80%	1.09 (0.83, 1.44)	0.523	1.21 (0.91, 1.62)	0.189
50–80%	1.26 (0.92, 1.71)	0.149	1.23 (0.90, 1.68)	0.195
<50%	1.00		1.00	
Urine test participation rate				
≥80%	0.65 (0.51, 0.83)	<0.001	0.78 (0.61, 1.01)	0.058
50–80%	1.04 (0.79, 1.36)	0.779	1.16 (0.88, 1.53)	0.306
<50%	1.00		1.00	
Urine positive rate				
≥60%	0.90 (0.74, 1.08)	0.252	0.95 (0.77, 1.17)	0.644
20–60%	1.20 (1.03, 1.40)	0.021	1.15 (0.97, 1.35)	0.099
<20%	1.00		1.00	
Length of follow-up intervals (month)	0.99 (0.97, 1.00)	0.132	1.00 (0.99, 1.02)	0.828

OR, odds ratio; CI, confidence interval. aOR, adjusted OR.
